# Asphalted Road Temperature Variations Due to Wind Turbine Cast Shadows

**DOI:** 10.3390/s91108863

**Published:** 2009-11-05

**Authors:** Rafael Arnay, Leopoldo Acosta, Marta Sigut, Jonay Toledo

**Affiliations:** Department of Systems Engineering and Automatics, University of La Laguna, Avda. Francisco Sánchez S/N, 38204 La Laguna, Canary Islands, Spain; E-Mails: leo@isaatc.ull.es (L.A.S); marta@isaatc.ull.es (M.S.S.); jonay@isaatc.ull.es (J.T.C.)

**Keywords:** shadow removal, road detection, thermal spectrum images

## Abstract

The contribution of this paper is a technique that in certain circumstances allows one to avoid the removal of dynamic shadows in the visible spectrum making use of images in the infrared spectrum. This technique emerged from a real problem concerning the autonomous navigation of a vehicle in a wind farm. In this environment, the dynamic shadows cast by the wind turbines' blades make it necessary to include a shadows removal stage in the preprocessing of the visible spectrum images in order to avoid the shadows being misclassified as obstacles. In the thermal images, dynamic shadows completely disappear, something that does not always occur in the visible spectrum, even when the preprocessing is executed. Thus, a fusion on thermal and visible bands is performed.

## 1. Introduction

*Verdino* is a self guided, electrical vehicle [1] that will carry out the autonomous transportation of passengers in a bioclimatic estate located in the Technological Institute of Renewable Energies (ITER) in the south of Tenerife, Canary Islands, Spain. The vision system of *Verdino* consists of three visible spectrum cameras and one infrared thermal spectrum camera. The detection of the road is accomplished by applying a variation of an Ant Colony Optimization metaheuristic described in Section 2. The input information for the road detection algorithm is a contribution of the information provided by one visible spectrum camera and the thermal one. The way the different image sources are used depends on certain environmental circumstances, explained in Section 4.

The ITER has a wind farm located in the surroundings of the route where the vehicle is going to navigate. This results in dynamic cast shadows produced by the wind turbines appearing on the road, making it harder to achieve a correct road detection in the visible spectrum.

In computer vision, cast shadows in an image frequently lead to misdetections. Preprocessing the image to remove these shadows is a common procedure that additionally loads the CPU. Consequently, the frame rate can drop under what is considered acceptable for real time detections. The study presented in this paper determines the variations of temperature in an asphalted road due to wind turbines' dynamic cast shadows and the conditions that lead to them. If the difference of temperature in the road surface between a shaded area and an irradiated one is relatively small, it will not be detected in the infrared spectrum. In these cases, the road detection will be entirely based on the infrared (thermal) spectrum, saving both the preprocessing time that would be needed to remove the shadows in the visible spectrum, and the problems in later processing as consequence of improper shadow elimination.

Thermal vision in the field of autonomous driving is frequently related to pedestrian detection [2]. The fusion of thermal and visible spectrum is well studied and implemented in satellite or aerial imagery. In [3] the infrared information is used to enhance airborne thematic mapper (ATM) data in cloud-shadowed areas. We have not found any other study in the literature that applies thermal vision to avoid the shadow-removal step.

This paper is organized as follows: Section 2 will describe the application of the Ant Colony Optimization algorithm to the vision problem, while in Section 3 a study of the impact of dynamic shadows on the road detection is performed. How the information from both visible and thermal spectrum images can be joined to overcome the shadows problem is explained in Section 4. Section 5 and Section 6 present some experimental results and conclusions, respectively.

## 2. Non-Structured Roads Detection Applying an Ant Colony Optimization (ACO) Algorithm

Lane detection in structured environments has been resolved using monochromatic stereo and monocular images in [4] and [5], respectively, using some *a priori* knowledge like the existence of line markers. These approaches can not be used on unknown, unstructured environments, due to the lack of such *a priori* information.

Other authors have addressed the problem of navigating a robot vehicle in non-structured environments using different methods. Some of them use pattern recognition techniques [6], or algorithms based on the HSI color space and 2D-spatial constraints [7]. Recently, a method based on the extrapolation of the characteristics of a road pattern (supposedly free of obstacles) to detect the road [8] has been proposed. Obviously, the pattern chosen determines the performance of this kind of methods, relying on some other tools (like laser based obstacle detectors) to perform this task.

**Figure 1.**
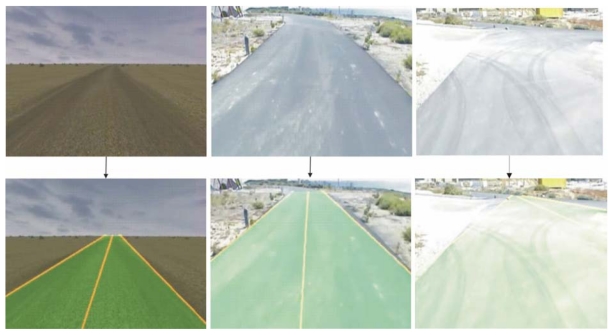
Application of the ACO algorithm to different types of non-structured roads input images: synthetic desert paths (left), unmarked roads (center) and partially occluded non-structured roads (right). The input of the algorithm is shown in the uppermost part of the figure and the output (detected road) is shown in the lowest part.

The approach proposed in this paper neither uses supplementary tools nor relies on the uniformity of the road to perform an extrapolation. It applies an Ant Colony Optimization to detect the road using information of the margins [9]. Due to degraded road boundaries, noise in the image and occlusion, among others, variable quality in the initial segmentation of the road can be achieved. Mapping the road detection problem as an optimization one allows the algorithm to perform well under different initial segmentation conditions and for different types of roads (Figure 1). Broggi and Cattani have also used the ACO methodology to detect roads [10]. In this paper, the initial segmentation process is different from the one they propose and the motion rule of the agents has been simplified, not implementing the backtracking process. Moreover, in the algorithm presented in this paper the offline pheromone contribution is influenced by a parameter proportional to the distance between the last pixel of the solution and the attraction point. The method has been generalized to indistinctly work with infrared and visible input images.

When the vehicle is on the road, it can be assumed that its margins are two curves that go from the bottom to the horizon line of the input image. Some local features of the pixels that conform these curves have to be found in order to establish a probability for these pixels to belong to them. The problem is then reformulated as an optimization one, so the ACO metaheuristic [11-15] can be applied. Pixels belonging to edges and located in a certain interest area that changes with the orientation of the road have a higher probability of belonging to the road margins. The optimal solutions are the ones containing the higher number of pixels belonging to the margins.

The next subsections resume some features of the ACO based road detection algorithm. For a complete description see [9].

### 2.1. Preprocessing

If necessary, a shadow removal step is performed (see Sections 4 and 5). Basically, the pattern of the road detected in the previous frame is used to look for low brightness areas. These areas are not taken into account in the *canny* edge detection procedure as they produce strong edges. Finally, some dilation and erosion operations are performed on the edge detection output in order to highlight long contours, theoretically belonging to the road margins.

### 2.2. Colony Initialization

The starting states of the agents will be located in the starting areas, where the colonies are going to be placed. These areas, with a high density of edge pixels and located in the laterals of the image, are, a priori, good starting points for the exploratory movement of the agents. They represent the start of the road margins as perceived in the image. Two colonies are placed on these areas, one to detect each margin.

The stop condition determines when the agents decide to stop their exploratory movement, obtaining a partial solution of the problem. This solution will be the path followed by the agents so far. When an agent reaches the row of pixels of the horizon line in the image, it stops.

### 2.3. The Point of Attraction

When an agent is in a given state, it can move to a group of feasible neighbors. These neighbours are calculated with what is called a point of attraction. Because the final objective of the algorithm is the real time road detection, the number of feasible neighbors to be explored has to be minimized. The point of attraction, located on the horizon line, where the supposed road boundaries intersect with each other, polarizes the movement of the agents. As a consequence of this, they tend to explore where the road limits are supposed to be.

### 2.4. The Motion Rule

The agents pass from their current states to the next ones following a movement rule that can be divided into two levels. The first level is the random-proportional rule for artificial ants proposed in the first ACO algorithm, Ant System. This rule determines a probability for each feasible neighbour of an agent of being its next position. This probability is influenced by heuristic information and the pheromone trails. The heuristic value of a pixel is directly proportional to its edge intensity. The second level is a pure random movement that is applied when there is no heuristic information and there are no pheromone trails either.

As seen, the artificial ants start in their initial states, defined by the starting areas located in the beginning of the road margins. They move row by row of pixels following their motion rule and building their solution until they reach the horizon line or, in other words, their stop condition.

### 2.5. Pheromone Update

When an agent reaches a stop condition, it updates the pixels that conform the path that it followed with artificial pheromone. The amount of pheromone the agent deposits is directly proportional to the number of edge pixels of the path, and inversely proportional to the distance between the last pixel of the path and the attraction point. Thus, the solutions that end nearer the attraction point are favored and the exploratory movement outside the road boundaries penalized.

### 2.6. Agents Organization

The colonies are divided into subgroups, each one of them with different motion rule parameters. The groups are sequentially executed. The first groups give more credit to the heuristic information, thus making an exploratory movement. However, the last groups are more likely to follow the pheromone trails. This is due to the fact that the pheromone information becomes more reliable as the agents update it with their solutions, which represent the accumulated experience of the colony.

### 2.7. Solution Extraction

The algorithm output consists of two paths, one for each road boundary. These paths are obtained by two special agents that are attracted by the pheromone trails only, travelling to the feasible neighbour with the greater amount of it.

### 2.8. Road Pattern Update

A very important step in the road detection process it is to determine the parameters of the road pattern. This pattern represents the road as the algorithm output. It is conformed by two lines, which are the result of the problem solution-paths interpolation. This simplified representation of the road (orientation of the road and the lateral distance to its margins) gives the required information for the control mechanism of the vehicle. The road pattern varies frame to frame with certain inertia, making rapid variations of the input information less harmful to the detection. Typically, these rapid variations are caused by noise in the image or potholes.

## 3. Study of the Effect of Wind Turbines Shadows on the Road Detection Performance

Cast shadows are a problem in vision-based road detection. In several articles this problem has been solved relying on some *a priori* knowledge of the road like, for example, visible lane markings [5]. The roads where *Verdino* is going to navigate are what it is called unstructured roads. This kind of roads has no lane markings or signals. Moreover, they do not have to be homogeneous or flat, so their detection can not be easily based on *a priori* knowledge [6-8].

When cast shadows appear during visible spectrum-based road detection, they can be misclassified as obstacles. If the shadows are on the road, they have to be removed in a preprocessing step in order to achieve a good road detection. Apart from the fact that this preprocessing step takes time, which is a problem from the real time road detection point of view, the main drawback is that the complete removal of the shadows is not guaranteed.

Cast shadows on the road cool the surface. If the shadow is static enough, the temperature of the shaded area starts to decrease, so a gradient of temperature between this area and a contiguous one that is irradiated by the sun appears. When this gradient surpasses certain value, the shadow is detected by both the visible and the thermal-based vision system as an obstacle. On the other hand, if the shadow is dynamic (as the ones cast by the wind turbines) there is less time for the asphalt to cool, so the gradient of temperature remains small.

As it will be explained in more detail in the next section, the gradient of temperature depends on the occlusion percentage due to the wind turbine blades and their spinning speed. In certain conditions, shadows that are perfectly perceived using a camera in the visible spectrum disappear when using the thermal camera. It is in these conditions when the road detection mechanism is entirely based on the thermal information so there is no need for a shadow removal step.

### 3.1. Asphalt Cooling due to Wind Turbine Dynamic Cast Shadows

As previously said, there is a wind farm in the surroundings where the vehicle is going to navigate. Due to wind turbines' cast shadows, certain regions of the road are periodically shaded. This periodicity depends on the wind turbine blades spinning speed. The slower the blades spin, the lower the temperature of the affected asphalt area is going to be, because it will be shaded for a longer period of time.

Any surface exposed to solar radiation absorbs part of this radiation. If the area of the surface is *A (m^2^)* and the amount of incident solar radiation is $I\;\left(\frac{w}{m^{2}}\right)$, the absorbed energy per unit of time is *aAI (w), a* being the solar absortance (0 ≤ *a* ≤ 1)that basically depends on the color of the surface. The darker the surface is, the bigger *a* is.

If the material has a thermal capacity $C=cm\;\left(\frac{J}{{}^{\circ}C}\right)$, being $c\;\left(\frac{J}{Kg{}^{\circ}C}\right)$ the specific heat of the material and *m (Kg)* its mass, then the increment of heat per unit of time is $\left(\frac{\text{CdT}}{dt}\right)$, *T* being the temperature. This increment must be equal to the absorbed energy per unit of time, as shown in equation 1:

(1) $$C\frac{dT}{dt}=\text{aAI}$$

According to this expression, given a constant solar irradiation, the temperature rises constantly without limit. However, when the temperature of the material rises above the ambient temperature *T_a_*, it losses heat towards the environment, via radiation, conduction or convection. This heat flow is proportional to the difference of temperature between the material and the ambient:

(2) $$AU_{l}\left(T-T_{a}\right)$$

$U_{1}\;\left(\frac{w}{{}^{\circ}\;Cm^{2}}\right)$ is the general losses coefficient, which contributes to the heating of the material with another term, as shown in equation 3:

(3) $$C\frac{dT}{dt}=\text{AaI}-AU_{l}\left(T-T_{a}\right)$$

The insolation that reaches the road surface can be decomposed into two different types: direct and diffuse. The diffuse insolation comes from several directions and is the result of the scattering and reflexion of the radiation due to atmospheric components in the sky. The estimation of the road temperature in a shaded area is mainly based on the amount of direct insolation that reaches the surface, as the diffuse one remains the same.

In their periodic movement, the shadows of the wind turbine blades cool the surface of the asphalt in the whole area *A (m^2^)* of the projection of the circle described by them. Let us call this the *projection area*. Given a direct insolation measurement $I_{d}\;\left(\frac{w}{m^{2}}\right)$, the amount of direct insolation that effectively reaches *A* is given by equation 4:

(4) $$I_{e}=I_{d}(1-p)$$

where *p* is the ratio between the area of the blades and the area of the circle described by them in their spinning movement. In other words, when the direct insolation gets through the wind turbine, it will be occluded by the blades in a proportion equal to the mentioned ratio. Let us call *p* the occlusion percentage.

As mentioned earlier, when a wind turbine spins, the reduction of irradiation distributes homogenously in the projection area. Two different situations must be taken into account in order to study the temperature gradients. On the one hand, when the wind turbine blades spinning speed is above a certain threshold, the individual cast shadows of the blades do not generate a temperature gradient detectable by the thermal camera, but they go on contributing to the cooling of the projection area. In Section 5, some results concerning the study of the gradient of temperature that appears between the projection area and the contiguous one is carried out. On the other hand, when the spinning speed is low, individual blades cast shadows generating a temperature gradient that is detected by the thermal camera because there is more time for the asphalt to cool. In Section 5 a spinning speed threshold for which a detectable gradient appears is established.

From the vision-based road detection point of view, these two kinds of situations can be misclassified as obstacles on the road. In the first case, the gradient between a projection area and a non-shaded area can be misclassified as a static obstacle on the road. In the second case, the gradients caused by the individual blades can be miss-classified as rapid moving obstacles.

### 3.2. Influence of the Wind Turbine Blades Spinning Speed

If the wind turbines' blades spinning speed is high enough, the temperature of the projection area decreases homogenously. The greater the occlusion percentage is, the faster the road surface cools.

**Figure 3.**
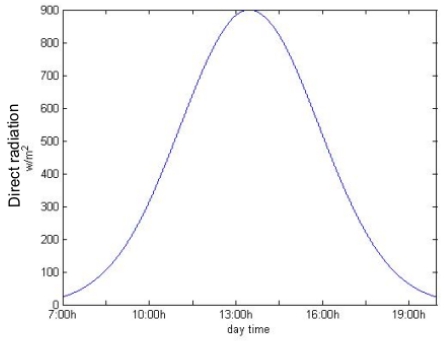
Direct radiation during a day in the ITER surroundings.

**Figure 4.**
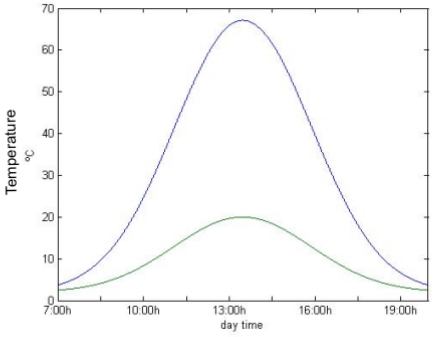
Asphalt (blue line) and external temperature (green line) during a day in the ITER surroundings.

Asphalt temperature can be estimated using equation 3. Experimental results show that both the ambient temperature behavior and radiation intensity variations during a day can be approximated by a Gaussian distribution. In Figures 3 and 4 the direct radiation and the asphalt and ambient temperatures during a sunny day in the ITER surroundings, respectively, are shown.

**Figure 5.**
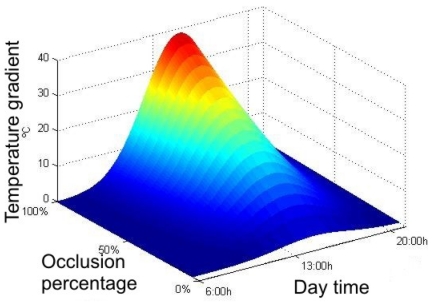
Temperature gradient between shaded and non shaded areas in proximity during a day for different occlusion percentages.

The model of the wind turbine determines the occlusion percentage due to the blades. This percentage modifies the direct radiation according to equation 4. In Figure 5, the gradients of temperature between a non-shaded area and a shaded one for a range of occlusion percentages are shown.

### 3.3. Reduction of the Shaded Area Temperature VS Blades Surface

**Figure 6.**
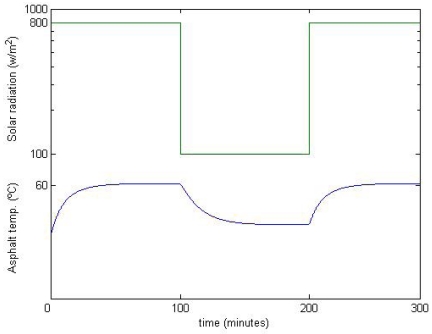
Asphalt temperature variation when exposed to periods of irradiation and shadows.

The temperature variation of a region of asphalt subject to a static shadow that blocks all the direct irradiation (100 % of occlusion) for a relatively long period of time is the one shown in Figure 6. In this figure, given an ambient temperature, it is shown how the temperature of the road surface (blue line) varies when it is exposed to periods of irradiation and shadows (green line).

The time needed for the shaded asphalt to cool *1 °C* is T_1c_ *(sec)*. Given a blade shadow width *l (m)*, there is a spinning speed $w_{1c}\;\left(\frac{\text{rad}}{\sec}\right)$ for which the shadows remain in the same place of the road the necessary time to cool it beneath a temperature which can be detected by the thermal camera. If *r* is the blade length, then the perimeter of the wind turbine is:

(5) P=2πr

The shadow width in radians is $\frac{l}{r}$, so it is necessary the shadow covers this width in *t* seconds as maximum for the asphalt to cool *1°C*. This gives a spinning speed shown in equation 6:

(6) $$W_{1{}^{\circ}C}=\frac{\overset{l}{\;}/\underset{r}{\;}}{T_{1{}^{\circ}C}}$$

**Figure 7.**
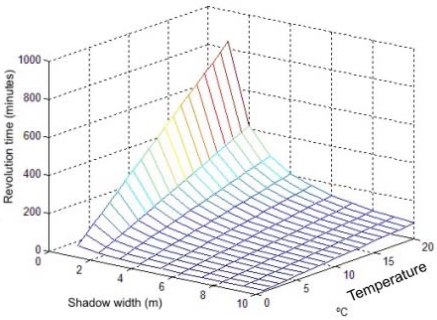
Minutes per revolution needed to obtain the temperature gradients showed for different shadow widths.

In Figure 7, the wind turbine blades revolution time expressed in minutes as a function of the cast shadows width and the temperature gradient between the shaded and non shaded areas is shown. As it can be seen, with an average shadow width of 1 m, more than three hours per revolution are needed for the asphalt to cool 5 °C. Wind turbines usually spin much faster. That is the reason why, in practice, the gradient of temperature is not noticed in the thermal spectrum.

## 4. Visible and Thermal Images Fusion to Solve the Shadows Problem

### 4.1. Visible or Thermal Images?

The information of the road obtained by processing images in the visible spectrum can be complemented by information obtained from images captured by a thermal camera. The ACO-based road detection algorithm described in Section 2 processes the two kinds of images, so the information obtained from both of them is joined to build a more accurate and robust representation of the road.

**Figure 8.**
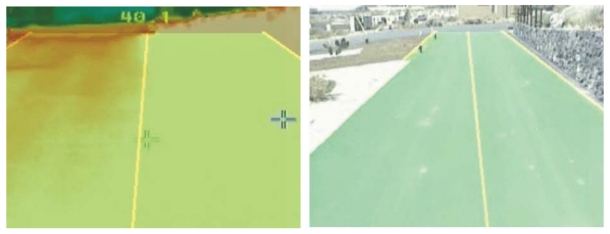
Both images in this figure show the same road stretch in the infrared (left) and visible (right) spectrums. As it can be seen in the visible spectrum image, there is a stone wall on the right margin of the road. The temperature of the wall is the same as the temperature of the road, making it impossible to distinguish the road from it margins in the thermal spectrum image. In cases like this, it is necessary to base the road detection on the visible spectrum input image only.

Due to the windy nature of the environment where the prototype is going to navigate, it is frequent for dust to partially cover the road, making it highly difficult to achieve a proper road detection. If the asphalt under the dust is warm enough, the temperature of the road will be considerably higher than the temperature of the margins. In these cases, the road can be detected by its temperature rather than by its visible borders. In certain illumination conditions, for example when facing the sun during dawn, the video sensor saturates, making it very difficult to achieve a proper road detection. In these situations the thermal vision may be used to complement the poor detection achieved when using the visible spectrum. Of course, this is valid when the temperature of the road is different than the temperature of the margins, in Figure 8 it is shown an example of the contrary case. This implies that the thermal images based detection will perform much better on sunny, warm days and, of course, during daytime. What is more, there are road stretches where the temperature measurements are very heterogeneous due to patches of different materials. In these zones it is easier to achieve a good detection via the visible spectrum.

**Figure 9.**
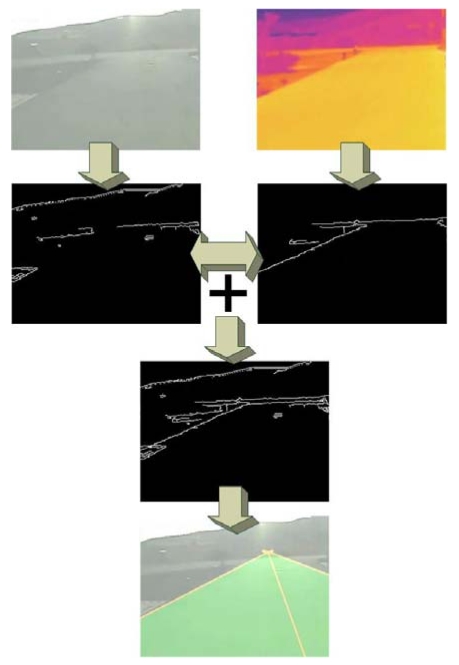
Example of how thermal (right) and visible (left) spectrum information is joined to improve the road detection.

When the previously mentioned illumination conditions are detected, the system starts capturing from two different video sources: one in the visible spectrum and another one in the thermal spectrum. In the first stages of the preprocessing step, right after the edge detection, both images are added, so the lack of edge information in the visible spectrum is complemented by the information obtained by the thermal camera, when possible. As seen, there is a time to use each kind of images, making it possible to complement the information from both to accomplish a proper road detection in a wide range of conditions, as shown in Figure 9.

### 4.2. Shadows Occluding the Road Margins

**Figure 10.**
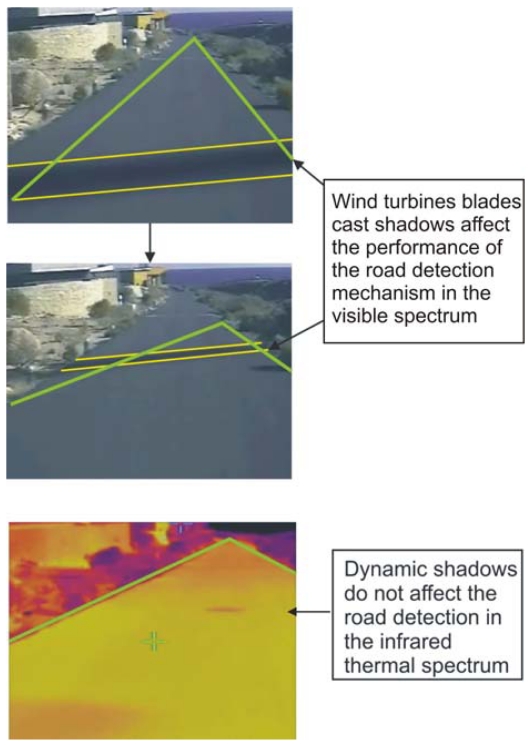
Example of how dynamic shadows difficult the road detection in the visible spectrum. However, they are usually not detected in the thermal spectrum.

Cast shadows on the road that are near its edges, like the ones produced by the wind turbines blades, affect the performance of the ACO-based road detection algorithm if they are not removed, as shown in Figure 10. Typically, cast shadows produce strong edges. If these edges are near the ones belonging to the road margins, the agents can miss the election and follow the shadow edge in their probabilistic exploratory movement. This can occur more frequently than desired because these edges inside the road will probably end near the point of attraction making them more appealing for the agents.

This situation is usually addressed by implementing a shadow removal step previous to the road detection. This step takes CPU time, which is very restricted in real time detection. The solution proposed in this article tries to anticipate these situations when difficult shadows produced by the wind turbines blades appear. Thus, the road detection switches the source image to work with the thermal ones, avoiding the shadow removal step.

## 5. Experimental Results

As it has been explained along the paper, shadows produce strong edges and, consequently, lead to misdetections if they are not removed. This makes it easy to detect them in the visible spectrum. On the other hand, shadows are not always detected in the thermal spectrum because they need to remain in the same position to cool the road under certain temperature.

The thermal camera assigns a color to each temperature detected in a scene. The wider the range of temperature in the scene is, the smaller the difference in the color code assigned to small temperature gradients is. This means that it is more difficult to detect small gradients when the range of temperature in a scene is big, since they produce weak edges in the preprocessing step. With this in mind, a *detectability* function can be defined to determine how well the temperature gradient is going to be detected as an edge in the preprocessing step. *Detectability* is defined as $\frac{d}{z}$ and it is in the range:

(7) $$0\leq \frac{d}{z}\leq 1$$

where *z* represents the range of temperature in a scene, in other words, the difference between the highest and the lowest temperatures detected. This range can be approximated by the difference between the irradiated asphalt temperature and the ambient one. *d* is the gradient of temperature between a shaded area and a non shaded one. When the ratio is big, the temperature gradient will be more probably detected and *vice versa*.

The *detectability* function on its own gives some idea of how well a gradient of temperature will be detected and segmented in the preprocessing step. However, it is necessary to establish some threshold above which it can be guaranteed the gradient will be detected.

To obtain the mentioned threshold, a set of grayscale images with increasing intensity difference between the background and a certain object were passed through a *canny*-based edge detection tuned as in the preprocessing step. Intensity differences from 14 to 255 grayscale levels make the object perfectly detectable by the edges processor. The minimum variation still detectable is 14 over 255, in other words, $\frac{14}{255}≃5.5\%$ of the grayscale range. Going back to the *detectability* function, the following inequality is obtained:

(8) d≥0.055∗z

**Figure 11.**
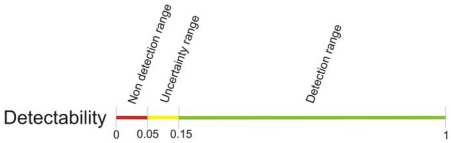
Thresholds for *detectability* range. Non detection range (red): temperature gradients are not detected in the preprocessing step. Uncertainty range (yellow): it can not be guaranteed if the gradient is detected or not. It strongly depends on the perspective in which the scene is captured. Detection range (green): the gradient is strong enough for being detected in the preprocessing step.

Because *z* is variable, an absolute threshold for *d* can not be defined. Instead of that, it can be designed as a percentage of *z*. If the variation of temperature between shaded and non shaded areas is a 5.5% or greater of the maximum variation in the scene, it will be detected in the thermal image preprocessing step. The obtained threshold can not be applied to the study as it is, though. In real road scenes, heat transfer occurs between shaded and non shaded areas in proximity. From the thermal vision point of view, given an Euclidean distance in pixels between two points in the image, the camera assigns a color gradient that depends on its *detectability*, as previously seen. However, the same temperature gradient on the road may reflect on different pixels sets on the image depending on the perspective in which the scene is captured, thus appearing sharper or smoother color gradients. That means that for a low range of *detectability* values, there is an uncertainty of the gradient being effectively detected. With this in mind, some tests were performed. On these tests, shaded and non-shaded patches of asphalt in proximity were recorded by the thermal camera. Empirical results show that, for relatively long road patches, it is needed a difference of at least 15% of the maximum temperature variation in the scene for being detected, as shown in Figure 11.

In the next sections, thresholds for the occlusion percentage and wind turbines spinning speed are obtained. Those thresholds are deduced from the ones for *detectability*, as shown in Figure 11. To validate the empirical results, a theoretical model of a road patch subject to periodic cast shadows has been developed. The periodic shadows are modeled as a square-wave signal where the period is proportional to the spinning speed, and the width of the pulse is proportional to the occlusion percentage of the wind turbine.

### 5.1. Spinning Speed Threshold

**Figure 12.**
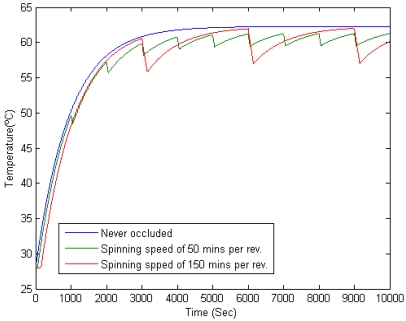
Temperature variation on a road patch surface due to periodic dynamic shadows. Green and red curves show temperature variations for a spinning speed of 50 and 150 minutes per revolution, respectively. Blue curve shows the temperature of a non-shaded road patch.

Spinning speed thresholds can be obtained from the ones on the *detectability* range. When the spinning speed of the wind turbines blades is above 50 minutes per revolution, the individual blades produce a temperature gradient. In Figure 12 this behavior is modeled for 800 *w/m^2^* of irradiation and 25 °C of ambient temperature, for an average wind turbine of 7% of occlusion, that is the average occlusion in the wind farm of the ITER. The uppermost threshold for the spinning speed (150 mins. per rev.) is showed in a red curve. As seen, ≃ 5 °C temperature variations due to periodic shadows are produced: this is the gradient between the individual blades shadows and an irradiated area in their proximity. The maximum gradient of temperature, that is to say, the difference between the highest and the ambient temperatures, in the graph is ≃ 34 °C. Then, *Detectability* is $\overset{d}{\;}/\underset{z}{\;}\to \overset{5}{\;}/\underset{34}{\;}≃0.15$, corresponding to the highest threshold, shown in Figure 11. The same correspondence can be obtained for the lowest threshold of spinning speed, showed in a green curve. Table 1 numerically shows those gradients for the lowest and the uppermost thresholds.

The relation between spinning speed and *detectability* is shown in Figure 14. As can be seen in ths figure, *detectability* grows inversely proportional to the spinning speed, while it remains independent of the time of the day. In windy conditions, wind turbines spins between 15 and 45 revolutions per minute, much faster that the lowest threshold obtained for the spinning speed, which is 50 m.p.r (50 minutes per revolution = 0.02 revolutions per minute). That is why in practice there is no noticeable gradient produced by the blades when the turbine is spinning. Only when it is almost stopped it can be detected.

### 5.2. Occlusion Threshold

**Figure 13.**
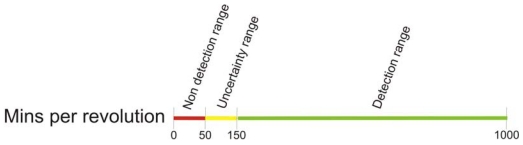
Thresholds for the wind turbines spinning speed range (m.p.r). Non detection range (red): individual blades gradients are not detected in the preprocessing step. Uncertainty range (yellow): it can not be guaranteed if the gradient is detected or not. It strongly depends on the perspective in which the scene is captured. Detection range (green): the gradient is strong enough for being detected in the preprocessing step.

**Figure 14.**
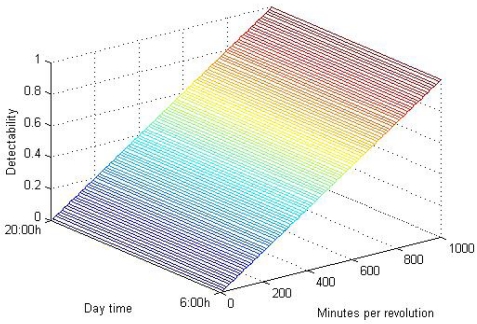
*Detectability* as function of the wind turbines blades revolution time.

**Figure 15.**
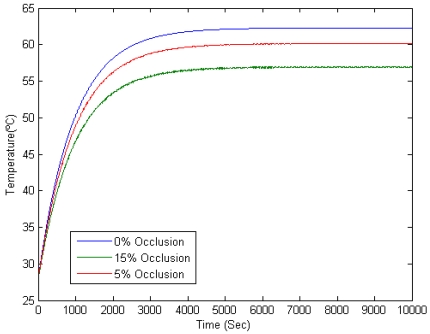
Temperature variation on a road patch surface due to periodic dynamic shadows for a spinning speed of 1 minute per revolution. Red and green curves show temperature variations for 5% and 15% of occlusion, respectively. Blue curve shows the reference temperature of a non-shaded road patch.

Occlusion percentage thresholds can be also obtained from the ones in the *detectability* range. When the spinning speed of the wind turbines is high enough (beneath 50 minutes per revolution; which is the lowest threshold for spinning speed obtained in the previous section, as shown in Figure 13), individual blades gradients are not detected in the preprocessing step. In these circumstances, the temperature of the road in the projection area homogeneously lowers according to a reduction of irradiation equivalent to the occlusion percentage. In Figure 15 this behavior is modeled for 800 *w/m*^2^ of irradiation and 25 °C of ambient temperature. The lowest threshold for the occlusion range (5 %) is shown in a red curve. As seen, temperature stabilizes approximately 2 °C beneath the maximum temperature of the road: this is the gradient between the projection area and an area in its proximity that is always irradiated. The maximum gradient of temperature in the graph is ≃ 34°C. Then, *Detectability* is d/z → 2/34 = 0.05, corresponding to the lowest threshold, shown in Figure 11. The same correspondence can be obtained for the highest threshold of occlusion, showed in a green curve. Table 2 numerically shows those gradients for the lowest and the uppermost thresholds.

**Figure 16.**
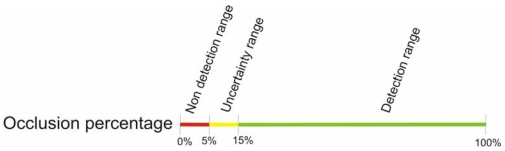
Thresholds for the occlusion percentage of the wind turbines. Non detection range (red): projection area gradient is not detected in the preprocessing step. Uncertainty range (yellow): it can not be guaranteed if the gradient is detected or not. It strongly depends on the perspective in which the scene is captured. Detection range (green): the gradient is strong enough for being detected in the preprocessing step.

**Figure 17.**
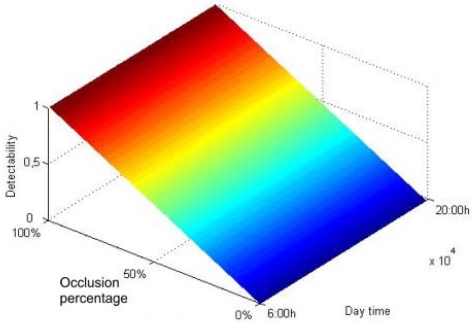
Detectability as a function of the occlusion percentage during a day.

As it can be seen in Figure 17, *detectability* grows with the occlusion percentage while it remains independent of the time of the day. Transferring the *detectability* thresholds to the occlusion percentage range, it is needed at least a 15% of occlusion to guarantee the detection of the gradient, as shown Figure 16. Considering that the average occlusion for the ITER wind turbines is 7%, it is very improbable for them to produce a noticeable difference of temperature, as it corresponds to the lowest part of the uncertainty range, see Figure 16. In practice, no gradient has been detected for this percentage of occlusion, although it cannot be theoretically discarded.

The relation between occlusion and *detectability* is showed in Figure 17.

## 6. Conclusions

Road detection in the presence of shadows is a problem that has been addressed by several authors. While the usual solution proposed consists on carrying out a shadows removal preprocessing stage, in this paper this problem is overcome by the use of a thermal camera.

The self-guided electrical vehicle *Verdino* is going to navigate in a route in whose near surroundings there is a wind farm. The shadows produced by the wind turbines blades can be wrongly classified as obstacles when visible spectrum images are used for the road detection. As it is well known, shadow removal procedures do not completely guarantee to eliminate the shaded areas, so they are both time consuming and imperfect. However, if the asphalt cooling due to the shadows is not big enough the shadows do not appear in the thermal images. The method proposed in this paper switches the road detection input information to thermal vision only when the proper conditions are given. In these conditions, it is guaranteed that the shadows are not detected in the thermal spectrum, saving both the time needed for the removal step and the problems in later processing as consequence of non-proper shadows elimination. This is the reason why in this paper a study of the influence on the shaded area temperature reduction of both the wind turbines blades spinning speed and the blades surface is presented. As seen in the experimental section, the conditions required for the shadows absence in the thermal images are satisfied in the ITER wind farm. Only the average occlusion percentage lies on the lowest part of the *detectability* uncertainty range. That means that it can be possible to detect a gradient in the preprocessing step, although this is very improbable. In practice it has not been detected. In spite of the advantage of working with thermal images from the point of view of dynamic shadows, the ACO algorithm implemented to carry out the road detection can not do without the visible spectrum cameras. As explained in Section 4, there exist certain situations in which it is more efficient to use the visible camera and others in which the thermal camera provide with more useful information for the autonomous navigation. As a consequence of this, the fusion of the visible and thermal images is the solution proposed in this paper to solve, in certain circumstances, the shadows problem in navigation based on vision.

## Figures and Tables

**Figure 2.**
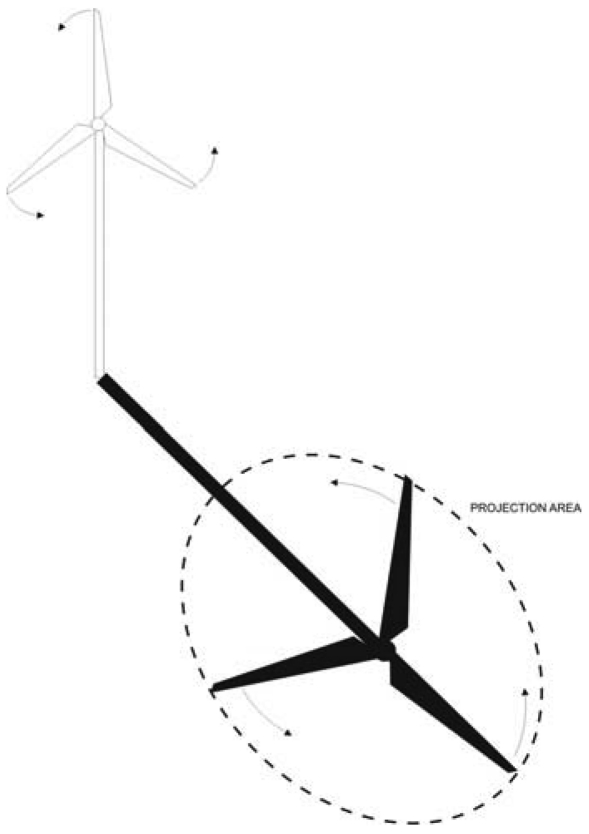
Direct radiation occluded by the wind turbine generates the *projection area*.

**Table 1.** Gradients of temperature produced by individual blade shadows corresponding to the proposed thresholds. (7 % occlusion, 800*w/m*^2^, 25°C ambient temp.).

**Spinning speed**	**Blade gradient min. temp.**	**Blade gradient max. temp.**	***d***	***z***	***Detectability***
50 m.p.r	59.37 °C	61.26 °C	1.89 °C	34.28 °C	0.0552
150 m.p.r	56.78 °C	61.95 °C	5.17 °C	34.28 °C	0.1508

**Table 2.** Gradients of temperature produced between the projection area and the irradiated area in its proximity corresponding to the proposed thresholds. (800 *w/m^2^*, 25°C ambient temp.).

**Occlusion %**	**Projection area gradient min. temp.**	**Projection area gradient max. temp.**	***d***	***z***	***Detectability***
5 %	60.27 °C	62.28 °C	2.11 °C	34.28 °C	0.058
15 %	57.00 °C	62.28 °C	5.28 °C	34.28 °C	0.154

## References

[1] Bertozzi M., Broggi A., Fascioli A. (2000). Vision-based Intelligent Vehicles: State of the Art and Perspectives. J. Robot. Aut. Syst..

[2] Bertozzi M., Broggi A., Gomez C.H., Gomez R.I., Frediga R.I., Vezzoni G., Del Rose M. (2007). Pedestrian Detection in Far Infrared Images Based on the Use of Probabilistic Templates.

[3] Lui J.G., Moore J.M. (1993). Cloud Shadow Suppression Technique for Enhancement of Airborne Thematic Mapper Imagery. Photogramm. Eng. Remote Sens..

[4] Bertozzi M., Broggi A. (1998). GOLD: A Parallel Real-Time Stereo Vision System for Generic Obstacle and Lane Detection. IEEE Trans. Image Proc..

[5] Broggi A. (1995). Robust Real-Time Lane and Road Detection in Critical Shadow Condition.

[6] Crisman J., Thorpe C. (1991). UNSCARF: A Color Vision System for the Detection of Unstructured Roads.

[7] Sotelo M.A., Rodrguez F.J., Magdalena L., Bergasa L.M., Boquete L. (2004). A Color-Based Lane Tracking System for Autonomous Driving on Unmarked Roads. Auto. Robots.

[8] Dahlkamp H., Kaehler A., Stavens D., Thrun S., Bradski G. (2006). Self-Supervised monocular Road Detection in Desert Terrain.

[9] Arnay R., Acosta L., Sigut M., Toledo J. (2009). Applying an Ant Colony Optimization Algorithm to an Artificial Vision Problem in a Robotic Vehicle. Adv. Soft Comput..

[10] Broggi A., Cattani S. (2006). An Agent Based Evolutionary Approach to Path Detection for Off-Road Vehicle Guidance. Pattern Recognition Lett..

[11] Denebourg J.L., Pasteels J.M., Vergaeghe J.C. (1983). Probabilistic Behaviour in Ants: A Strategy of Errors?. J. Theor. Biology.

[12] Dorigo M., Stützle T. (2002). The Ant Colony Optimization Metaheuristic: Algorithms, Applications and Advances. Handbook of Metaheuristics.

[13] Dorigo M., Stützle T. (2004). Ant Colony Optimization.

[14] Dorigo M., Maniezo V., Colorni A. (1996). The Ant System: Optimization by a Colony of Cooperating Agents. IEEE Trans. Syst. Man Cybern..

[15] Heck P.S., Ghosh S. (1996). A Study of Synthetic Creativity: Behavior Modeling and Simulation of An Ant Colony. IEEE Int. Syst..

